# Increased urinary CD80 excretion and podocyturia in Fabry disease

**DOI:** 10.1186/s12967-016-1049-8

**Published:** 2016-10-13

**Authors:** H. Trimarchi, R. Canzonieri, A. Schiel, C. Costales-Collaguazo, J. Politei, A. Stern, M. Paulero, T. Rengel, J. Andrews, M. Forrester, M. Lombi, V. Pomeranz, R. Iriarte, A. Muryan, E. Zotta, M. D. Sanchez-Niño, A. Ortiz

**Affiliations:** 1 Nephrology Service, Hospital Británico de Buenos Aires, Perdriel 74, 1280 Buenos Aires, Argentina; 2 Central Laboratory, Hospital Británico de Buenos Aires, Buenos Aires, Argentina; 3 IFIBIO Houssay, CONICET, Physiopathology, Pharmacy and Biochemistry Faculty, Universidad de Buenos Aires, Buenos Aires, Argentina; 4 Neurology Department, Laboratorio Neuroquímica Dr. Néstor Chamoles, Buenos Aires, Argentina; 5 IIS-Fundacion Jimenez Diaz, School of Medicine, UAM, Avda Reyes Catolicos 2, 28040 Madrid, Spain; 6 REDINREN, Madrid, Spain

**Keywords:** Enzyme replacement therapy, Fabry disease, Lyso-Gb3, Podocyte, Podocyturia, CD80, Proteinuria

## Abstract

**Background:**

Certain glomerulopathies are associated with increased levels of CD80 (B7-1). We measured the urinary excretion of CD80, podocyturia and proteinuria in controls and in subjects with Fabry disease either untreated or on enzyme replacement therapy (ERT).

**Methods:**

Cross-sectional study including 65 individuals: controls (n = 20) and Fabry patients (n = 45, 23 of them not on ERT and 22 on ERT). Variables included age, gender, urinary protein/creatinine ratio (UPCR), estimated glomerular filtration rate (eGFR), urinary uCD80/creatinine ratio (uCD80) and podocyturia. CD80 mRNA expression in response to lyso-Gb3, a bioactive glycolipid accumulated in Fabry disease, was studied in cultured human podocytes.

**Results:**

Controls and Fabry patients did not differ in age, eGFR and gender. However, UPCR, uCD80 and podocyturia were significantly higher in Fabry patients than in controls. As expected, Fabry patients not on ERT were younger and a higher percentage were females. Non-ERT Fabry patients had less advanced kidney disease than ERT Fabry patients: UPCR was lower and eGFR higher, but uCD80 and podocyturia did not differ between non-ERT or ERT Fabry patients. There was a significant correlation between uCD80 and UPCR in the whole population (r 0.44, p 0.0005) and in Fabry patients (r 0.42, p 0.0046). Lyso-Gb3 at concentrations found in the circulation of Fabry patients increased uCD80 expression in cultured podocytes.

**Conclusions:**

Fabry disease is characterized by early occurrence of increased uCD80 excretion that appears to be a consequence of glycolipid accumulation. The potential for uCD80 excretion to reflect early, subclinical renal Fabry involvement should be further studied.

## Background

Fabry disease is an X-linked storage disease due to mutations in the GLA gene encoding α-galactosidase A, leading to the lysosomal accumulation of enzyme substrates, namely globotriaosylceramide (Gb3), lyso-globotriaosylceramide (lyso-Gb3) and galabiosylceramide [1]. The intracellular pathological overload of these glycophingolipids disturbs cell morphology and leads to cell dysfunction [2–4]. Gb3 is the best-known metabolite and accumulates mostly inside cells. However, in the extracellular space, Gb3 circulates inside lipoproteins and the concentration in Fabry males is only 2- to 4-fold above control values [5]. By contrast, lyso-Gb3, a more hydrosoluble derivative of Gb3, circulates at concentrations 200-to 500-fold above normal values [5]. The precise mechanisms by which these metabolites lead to cell dysfunction remain elusive. It has been speculated that the interaction of glycosphingolipids with ion channels or transporters, mainly localized within the endoplasmic reticulum, or the activation of inflammatory pathways may contribute to cell and tissue damage [6–8]. Thus, Gb3 levels correlated with oxidative stress and inflammation in Fabry patients [9, 10]. Similar to diabetic nephropathy, Fabry nephropathy is a progressive proteinuric nephropathy of metabolic origin with a natural history that expands decades. In this regard, some glycolipids accumulated in Fabry disease, such as lyso-Gb3, are bioactive. Lyso-Gb3, but not Gb3, promoted the proliferation of vascular smooth muscle cells [11]. Moreover, lyso-Gb3 may activate inflammation pathways in renal cells [12]. Transforming growth factor-β1 (TGF-β1), Vascular endothelial growth factor (VEGF), Fibroblast growth factor-2 (FGF-2)—among others- are elevated in Fabry nephropathy, whilst Notch-1 signaling as well as apoptosis and autophagy-related inflammatory pathways are involved as well [13–16]. Specifically, at concentrations found in the circulation of Fabry patients, lyso-G3 promotes autocrine TGF-β1 and Notch-1 signaling in podocytes, a response that mimics podocyte responses to high glucose concentrations [14, 17]. High glucose also increases CD80 expression in cultured podocytes [18]. Lymphocyte activation antigen 7-1, also known as CD80, is normally located on antigen presenting cells as neutrophils, macrophages and dendritic cells. CD80 modulates CD4+ and CD8+ T cell activity by interacting with the co-stimulator ligand CD28 or with cytotoxic T-lymphocyte protein 4 (CTLA-4) [19]. Normal podocytes do not express CD80. In certain glomerulopathies, including diabetic nephropathy, CD80 is expressed by podocytes and tubular cells and the renal excretion is increased [18, 20]. Up-regulation of CD80 on podocytes and tubular cells under abnormal conditions suggests that these cells may behave as antigen-presenting cells and participate in inflammatory pathways [21]. Finally, CD80 has also been related to podocyte migration, detachment and podocyturia through its interaction with integrins. Podocyturia has been observed in active glomerulopathies [22, 23]. We have recently demonstrated the existence of podocyturia in Fabry disease, even in the absence of clinical nephropathy [24].

In this study, we explored the urinary excretion of CD80 in patients with Fabry disease and the potential drivers of CD80 expression.

## Methods

This is cross-sectional, observational study included 65 individuals. A group of 20 healthy subjects without known clinical morbidities or pharmacological treatment was recruited among potential kidney donors and subjects with normal laboratory results and clinical history. In addition, 45 Fabry patients were studied. Of them 23 were not treated with enzyme replacement therapy (ERT) while 22 had received ERT for at least 12 months with agalsidase beta 1 mg/kg administered every fortnight (Fabrazyme, Genzyme Corp, Cambridge, MA, USA). Fabry disease was diagnosed in all cases by low enzymatic alpha galactosidase A activity in dried blood spots and peripheral blood leukocytes, and confirmed by the identification of a GLA gene mutation. Patient characteristics are outlined in Table 1. The following variables were studied: age, gender, glomerular filtration rate estimated (eGFR) by the Chronic Kidney Disease-Epidemiology Collaboration equation (CKD-EPI), urinary protein-creatinine ratio (UPCR), podocyturia adjusted per gram of creatininuria and urinary CD80/creatinine ratio (uCD80).

**Table 1:** General characteristics of controls and Fabry patients

Variables	Controls (n: 20)	Fabry (n: 45)	p value
Age (years)	30 (20–48)	33 (11–86)	0.92
Gender (males)	10 (50 %)	18 (40 %)	0.63
eGFR (ml/min/1.73 m^2^)	110 (86–141)	125 (21–165)	0.14
UPCR (g/g)	0.03 (0.02–0.27)	0.07 (0.02–5.68)	*0.01*
Podocyturia/Cr (cells/g)	4 (0–90)	32 (0–439)	*<0.001*
uCD80/Cr (ng/g)	17 (0–142)	45 (0–2966)	*0.04*

Significant values (p < 0.05)

### Podocyturia

We have previously described the method to study podocyturia in detail [24]. Briefly, a mid-stream freshly voided urine sample was collected on-site after a minimum of 3 h without voiding; and 20 ml of urine were centrifuged at 700*g* for 5 min in a cytospin. The supernatant was discarded and the sediment was stored in 100 µl aliquots at room temperature mixed with a 1.5 ml solution of 40 % formaldehyde diluted in phosphate-buffered saline (PBS) (pH 7.2–7.4) to reach a final 10 % concentration. Podocyte nuclei were stained with 40,6-diamidino-2-phenylindole (DAPI). Podocytes were identified by immunofluorescence using anti-synaptopodin as the primary antibody (1:100, Abcam, Cambridge, MA, USA) and IgG anti-rabbit Alexa Fluor^®^ 488 (1:100, Abcam, Cambridge, MA, USA) as the secondary antibody. Samples were analyzed employing an epifluorescent microscope. Following our standardized technique, podocytes were counted in 10 randomly chosen 20× fields and the average of the counted podocytes in the microscopy fields was considered as the final count for each subject. Results were corrected based on the urinary creatinine concentration of the initial urinary volume of 20 ml employed for podocyte counting [24, 25].

### CD80 and other determinations

Serum creatinine was assessed the same week that urine was collected for podocyte counting. UPCR was measured from the specimen employed for podocyte assessment. The urinary concentration of CD80 was determined employing a Human CD80 Instant ELISA (Instant ELISA^®^, affymetrix eBioscience, San Diego, CA USA).

### Ethics

The present protocol was approved by the Institutional Review Board of the Hospital Británico de Buenos Aires, Buenos Aires, Argentina. Informed consent was obtained from each study participant.

### Cell culture and reagents

Human podocytes are an immortalized cell line transfected with a temperature-sensitive SV40 gene construct and a gene encoding the catalytic domain of human telomerase [14, 17]. At a permissive temperature of 33 °C, cells remain in an undifferentiated proliferative state and divide. Raising the temperature to 37 °C results in growth arrest and differentiation to the parental podocyte phenotype. Undifferentiated podocyte cultures were maintained at 33 °C in RPMI 1640 medium with penicillin, streptomycin, ITS (insulin, transferrin, selenite), and 10 % FCS. Once cells reached 70–80 % confluence, they were fully differentiated by culture at 37 °C for at least 14 days [14, 17]. Cells were cultured in serum-free media 24 h prior to addition of stimuli and throughout the experiment. Lyso-Gb3 (Sigma, St. Louis, MO) was used at a concentration of 100 nM and tested negative for lipopolysaccharide. This concentration is clinically relevant, since circulating lyso-Gb3 has been reported to be in the 10–50 nM range for heterozygous females and above 100 nM in males [11]. Lyso-Gb3 was chosen as a stimulus among the diverse glycolipids that accumulate in Fabry disease, because in relative terms its extracellular concentration is much higher (one order of magnitude higher) in Fabry patients than in controls than Gb3. Additionally, there is evidence that, unlike Gb3, lyso-Gb3 is bioactive in diverse cell systems, including vascular smooth muscle cells and podocytes [11, 14, 17]. In this regard, the 100 nM concentration of lyso-Gb3 was previously shown to be bioactive in cultured human podocytes in dose response studies [14, 17].

### Real time reverse transcription-polymerase chain reaction

RNA was isolated using Trizol reagent (Invitrogen, Paisley, UK). One microgram RNA was reverse transcribed with High Capacity cDNA Archive Kit (Applied Biosystems, Foster City, CA). Real-time PCR reactions were performed on the ABI Prism 7500 sequence detection PCR system (Applied Biosystems) according to the manufacturer’s protocol using the DeltaDelta Ct method [14, 17]. Expression levels are given as ratios to GAPDH. Pre-developed primer and probe assays were from Applied Biosystems.

### Immunohistochemistry

Kidney samples were obtained from excess tissue corresponding to kidney nephrectomy specimens donated to the biobank of the IIS-Fundacion Jimenez Diaz Biobank after diagnostic evaluation was performed. The local Ethics Committee approved the study protocol and informed consent was obtained. Control human kidney specimens were taken from normal portions of renal tissue from patients who underwent surgery because of localized renal tumors. One renal biopsy from a male Fabry patient was studied, a 69 year old with serum creatinine 4.2 mg/dl, proteinuria 0.7 g/24 h. Immunohistochemistry was carried out in paraffin-embedded tissue section 5 μm thick. The primary antibody was mouse monoclonal anti-CD80 (1:100, Abcam). Sections were counterstained with Carazzi’s hematoxylin. Negative controls included incubation with a non-specific immunoglobulin of the same isotype as the primary antibody.

### Statistical analysis

Results are expressed as median and ranges. Variables were analyzed using the Wilcoxon Mann–Whitney test. Correlations between variables were obtained with the Spearman correlation coefficient. Results were considered significant when p < 0.05. The statistical program employed was InfoStat 2016, Córdoba Argentina.

## Results

### Fabry patient studies

Controls and Fabry patients did not diff in age, eGFR or gender. However, UPCR, uCD80 and podocyturia were significantly higher in Fabry patients (Table 1). Fabry patients not on ERT were younger and there was a higher proportion of females. This is expected because both younger patients and females usually have a milder disease and this may underlie the decision not to initiate ERT yet. In this regard, eGFR was higher and UPCR lower in patients not on ERT, probably reflecting milder kidney disease. However, there were no differences in uCD80 excretion or podocyturia between Fabry patients on ERT and not on ERT (Table 2). There was a significant correlation between uCD80 and UPCR in the whole population (r 0.44, p 0.0005) and in Fabry patients (r 0.42, p 0.0046). However, there was no correlation between uCD80 and podocyturia.

**Table 2:** Intragroup comparison of Fabry patients

Variables	No ERT (n: 23)	ERT (n: 22)	p value
Age (years)	19 (11–75)	38.5 (17–86)	*0.02*
Gender (males)	4 (14 %)	14 (64 %)	*0.01*
Time on ERT (months)	0	42 (34–50)	*<0.0001*
eGFR (ml/min/1.73 m^2^)	141 (60–165)	120.5 (21–148)	*0.03*
UPCR (g/g)	0.06 (0.02–2.35)	0.11 (0.02–5.68)	*0.04*
Podocyturia/Cr (cells/g)	34 (4–439)	24 (0–107)	0.45
uCD80/Cr (ng/g)	40 (0–235)	47 (0–2966)	0.7
Mutations	D33G, L415P, R227X, A292T, N34D, C801, C326, C647A, C281, G640C, C1244T	D33G, L415P, R227X, A292T, N34D, D264Y, D155H, L180F	

Significant values (p < 0.05)

**Fig. 1:**
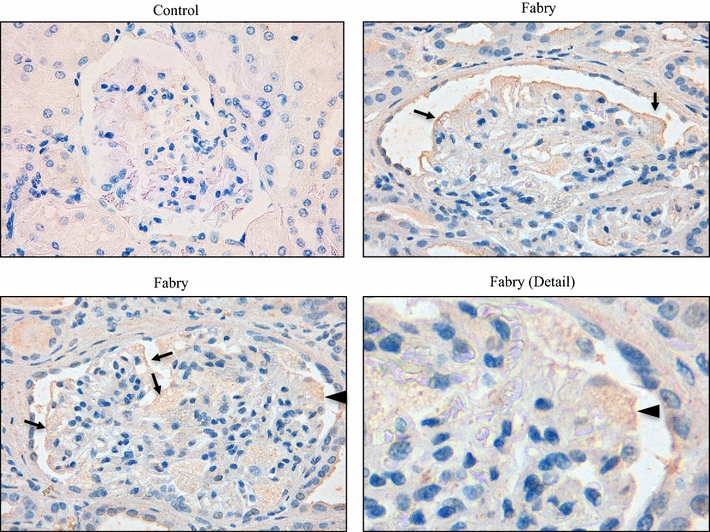
Expression of CD80 in human Fabry disease. Immunohistochemistry of a human Fabry biopsy shows increased expression of CD80 in podocytes (*arrows*). *Arrowhead* indicates the vacuolated, enlarged and CD80 stained podocyte observed in the detail. No staining was found in human control biopsies. Original magnification ×20 and ×40

Since uCD80 was increased in the urine of Fabry patients, we next explored the expression of CD80 in human Fabry disease tissue. No expression of CD80 was observed in control kidneys. However, immunohistochemistry confirmed CD80 expression in podocytes in a kidney biopsy from a Fabry patient (Fig. 1).

### Lyso-Gb3 increases CD80 expression in cultured podocytes

**Fig. 2:**
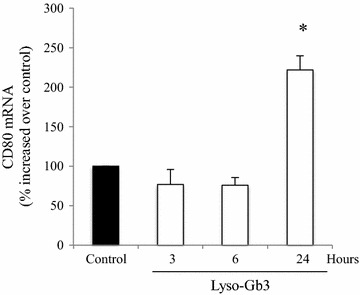
Lyso-Gb3 upregulates CD80 mRNA expression in podocytes. Cultured human podocytes were stimulated with 100 nM lyso-Gb3. Time-course of CD80 mRNA induction. *p < 0.004 vs control. Expression of mRNA was assessed by real time RT-PCR. Mean ± SEM of three independent experiments

Since uCD80 was increased early in Fabry nephropathy, even in the group of patients with better preserved eGFR and lower albuminuria, we explored whether glycolipids accumulated in Fabry disease may increase CD80 expression in kidney cells. We had previously shown that lyso-Gb3, at concentrations found in the circulation of Fabry disease patients, increases the expression of diverse mediators of kidney injury in a time- and dose-dependent fashion, with peak response observed at 100 nM and 24 h [14, 17]. Lyso-Gb3 at the concentration of 100 nM induced an increase in the mRNA expression of CD80 that peaked at 24 h in podocytes (Fig. 2).

## Discussion

In the present study, we have confirmed that in Fabry disease podocytes are lost in urine in higher amounts than in controls and that this is an early feature, present already in patients with subclinical kidney disease. If maintained over time, this may lead to podocyte loss and to the classical glomerular lesion of Fabry nephropathy: focal and segmental glomerulosclerosis. A novel finding is that uCD80 excretion is also increased early in Fabry nephropathy. While there was a correlation between uCD80 and proteinuria, the lack of correlation between uCD80 and podocyturia suggests that podocyturia appears not to be linked to the activation of CD80 signaling pathways and that uCD80 and podocyturia reflect different aspects of kidney injury in Fabry nephropathy. Moreover, we have identified lyso-Gb3 as a potential driver of CD80 expression in podocytes. Lyso-Gb3 had been previously shown to increase the expression or secretion of TGFβ1, CD74, Notch1 and MCP-1 in podocytes [14, 17]. Thus, lyso-Gb3 appears to promote a generalized stress response that we have now shown to also include CD80 and that is reminiscent of high glucose-elicited podocyte responses [18, 26, 27]. In this regard, another glycolipid, bacterial lipopolysaccharide, also increases CD80 expression in podocytes [28].

In Fabry disease, silent and heavier podocyturia occurred in younger individuals when median eGFR and proteinuria were still normal. Furthermore, uCD80 was already elevated in younger patients and females that were not yet on ERT, suggesting that it may be an early marker of kidney injury. In addition, uCD80 remained increased in patients on ERT. Since this was a cross-sectional study, it is unknown whether uCD80 had been higher at the start of ERT and had decreased but not normalized with ERT. Whether earlier therapeutic interventions could normalize uCD80 levels requires further investigations. The current literature remarks that in Fabry disease, early ERT initiation is associated to better long-term results [29, 30]. However, our cell culture data are consistent with the uCD80 observation, since lyso-Gb3 is decreased but usually not normalized by ERT [11].

Fabry nephropathy is a glomerulopathy. Thus, it is not surprising that the metabolic defect triggers the expression of inflammatory and fibrotic molecules, growth factors, cytokines and chemokines, as is the case for other glomerulopathies [31, 32]. In addition, a characteristic of Fabry nephropathy is that besides podocytes, endothelial and tubular cells accumulate glycolipids. These cells are all potential sources of inflammatory mediators. However, we were unable to measure simultaneously CD80 in blood and in urine and, thus, cannot determine whether uCD80 is of kidney origin. Notwithstanding these observations, our finding underscores the concept that in Fabry nephropathy, inflammatory molecules are excreted in urine and this appears not to be normalized by ERT. CD80 is not exclusive to lymphocytes, as podocytes in primary focal and segmental glomerulosclerosis [33] and diabetes [18] and podocytes and tubular cells in IgA nephropathy express CD80 in human biopsies [20]. Although some authors have remarked a potential role of CD80 in certain glomerulopathies, such as IgA nephropathy, primary focal and segmental glomerulonephritis and crescentic glomerulopathies [20, 21, 33, 34], others have recently seriously questioned these findings, particularly in minimal change disease and primary focal and segmental glomerulosclerosis [35]. Recent data point to glycogen synthase kinase (GSK) 3 as a key player in the pathogenesis of podocytopathy and proteinuria. In GSK3β knockout mice, de novo podocyte expression of nuclear factor kappa B (NFκB)-dependent mediators of podocyte injury, including CD80, cathepsin L, and MCP-1, was reduced [36]. These molecules may contribute to the pathophysiology of Fabry disease [14, 16, 37]. In Fabry disease, kidney cell CD80 expression may be driven by accumulated glycolipids, as shown in our cell culture experiments, and thus, it may provide information about continuing kidney injury by glycolipids despite ERT. Additional cytokines in the local microenvironment may also contribute.

It is currently unknown whether podocyte CD80 expression contributes to the pathogenesis of Fabry nephropathy by altering the shape, rearranging actin and altering glomerular permeability thus promoting proteinuria [21, 34]. Indeed, CD80 has been implicated in podocyte contraction, migration and eventual podocyte loss [21, 34]. However, we did not find a correlation between uCD80 and podocyturia. This may be related to small sample size. Alternatively, uCD80 and podocyturia may represent different aspects of Fabry nephropathy. Abnormal podocyte CD80 up-regulation has been linked to β1 integrin subunit abnormalities [21, 34, 38]. In this regard, increased urinary excretion of β3 integrin was increased in Fabry patients [38].

Since upregulated podocyte CD80 expression has been observed in several glomerulopathies, increased uCD80 levels are not expected to be diagnostic of Fabry nephropathy. Thus, they cannot presently replace renal biopsy. At present, assessment of podocyturia should be considered an experimental procedure. While it is a promising technique, since podocyte turnover is very limited and loss of podocytes results in irreversible glomerular injury, methods are not standardized for routine clinical assessment. It is likely that podocyturia is influenced by different factors, including availability of podocytes (it may decrease with advanced kidney disease due to loss of glomeruli) and rate of injury or death of remaining podocytes. In this regard, molecules expressed in stressed podocytes are likely shed and be present in urine as a result, without necessarily implying the urinary excretion of the podocyte of origin.

There are several limitations in our study: In absolute terms, the number of patients is small and the study is cross-sectional. However, few studies in rare renal diseases are able to recruit higher number of patients, and a prospective study will require several years of follow-up, given the 40 year natural history of Fabry nephropathy. We have not directly addressed the cellular source of CD80 in vivo in the studied patients. Whether the uCD80 originates from lymphocytes, neutrophils and/or dendritic cells, or from abnormal CD80 expression in podocytes, tubular or endothelial cells is unknown. Our findings are based on a highly sensitive and specific human ELISA, but are limited to urine, and we cannot discern whether there are differences in the behavior of circulating and urinary CD80 levels. However, an unrelated Fabry kidney biopsy and cell culture data suggest that podocytes could be one of the sources of uCD80. While some patients were off ERT and others on ERT, the cross sectional nature of the study does not allow to draw definitive conclusions on the role of podocyturia and excretion of CD80 as treatment monitoring biomarkers. The off ERT and on ERT groups are not comparable. Patients not yet on ERT were significantly younger and we have only one assessment for patients on ERT: in the absence of baseline values, it is unknown how these parameters might have changed upon initiation of ERT.

In summary, we confirmed the early development of podocyturia in Fabry patients. Moreover, we observed an early increase in uCD80. The fact that CD80 expression is increased by lyso-Gb3 in podocytes suggests that uCD80 may be an early marker of kidney injury in Fabry disease and also a marker of continued renal injury by glycolipids and future studies exploring the potential of uCD80 to reflect early, subclinical renal Fabry involvement are warranted.

## Conclusions

In Fabry disease podocyturia and urinary excretion of CD80 rise early in the disease process, even in patients with normal renal function. CD80 expression appears to be driven by exposure to glycolipids such as lyso-Gb3. This suggests that podocyturia and urinary CD80 expression may provide information into early and continuing kidney injury in Fabry disease. This hypothesis should be confirmed in larger longitudinal studies.

## Abbreviations

CD74: cluster of differentiation 74
CD80 (B7-1): lymphocyte activation antigen 7-1
CKD-EPI: chronic kidney disease-epidemiology collaboration
CTLA-4: cytotoxic T-lymphocyte protein 4
DAPI: 40,6-diamidino-2-phenylindole
eGFR: estimated glomerular filtration rate
ERT: enzyme replacement therapy
FGF-2: fibroblast growth factor-2
GAPDH: glyceraldehyde 3-phosphate dehydrogenase
Gb3: globotriaosylceramide
GLA: galactosidase alpha
GSK 3: glycogen synthase kinase
ITS: insulin, transferrin, selenite
Lyso-Gb3: lyso-globotriaosylceramide
MCP1: monocyte chemoattractant protein-1
NFκB: nuclear factor kappa B
PBS: phosphate-buffered saline
PCR: polymerase chain reaction
RNA: ribonucleic acid
TGF-β1: transforming growth factor-β1
UPCR: urinary protein/creatinine ratio
VEGF: vascular endothelial growth factor
